# Oxidation Kinetics of Ti-6Al-4V Alloys by Conventional and Electron Beam Additive Manufacturing

**DOI:** 10.3390/ma16031187

**Published:** 2023-01-30

**Authors:** Francisco Estupinán-López, Carlos Orquiz-Muela, Citlalli Gaona-Tiburcio, Jose Cabral-Miramontes, Raul German Bautista-Margulis, Demetrio Nieves-Mendoza, Erick Maldonado-Bandala, Facundo Almeraya-Calderón, Amit Joe Lopes

**Affiliations:** 1 Centro de Investigación e Innovación en Ingeniería Aeronáutica (CIIIA), FIME, Universidad Autónoma de Nuevo León, San Nicolás de los Garza 66455, Mexico; 2 W. M. Keck Center for 3D Innovation, The University of Texas at El Paso, El Paso, TX 79968, USA; 3 División Académica de Ciencias Biológicas, Universidad Juárez Autónoma de Tabasco, Villahermosa 86040, Mexico; 4 Facultad de Ingeniería Civil, Universidad Veracruzana, Xalapa 91000, Mexico

**Keywords:** titanium, additive manufacturing, oxidation kinetics, electron beam fusion

## Abstract

New manufacturing processes for metal parts such as additive manufacturing (AM) provide a technological development for the aeronautical and aerospace industries, since these AM processes are a means to reduce the weight of the parts, which generate cost savings. AM techniques such as Laser Powder Bed Fusions (LPBF) and Electron Beam Fusion (EBM), provided an improvement in mechanical properties, corrosion resistance, and thermal stability at temperatures below 400 °C, in comparison to conventional methods. This research aimed to study the oxidation kinetics of Ti-6Al-4V alloys by conventional and Electron Beam Additive Manufacturing. The thermogravimetric analysis was performed at temperatures of 600 °C, 800 °C, and 900 °C, having a heating rate of 25 °C/min and oxidation time of 24 h. The microstructural analysis was carried out by thermogravimetric analysis. Thickness and morphology of oxide layers were analyzed by field emission scanning electron microscope, phase identification (before and after the oxidation process) was realized by X-ray diffraction at room temperature and hardness measurements were made in cross section. Results indicated that the oxidation kinetics of Ti-6Al-4V alloys fabricated by EBM was similar to conventional processing and obeyed a parabolic or quasi-parabolic kinetics. The samples oxidized at 600 °C for 24 h presented the lowest hardness values (from 350 to 470 HV). At oxidation temperatures of 800 and 900 °C, however, highest hardness values (from 870 close to the alpha-case interface up to 300 HV in base metal) were found on the surface and gradually decreased towards the center of the base alloy. This may be explained by different microstructures presented in the manufacturing processes.

## 1. Introduction

In the aeronautics and aerospace industries, titanium alloys are used because of their very high specific mechanical properties, low density and their corrosion resistance at low temperatures [1]. One of the most important alloys in the Alfa-Beta category is the Ti-6Al-4V alloy, which has greatly influenced applications in the military, chemical, biomedical, petrochemical, and automotive industries [1,2,3], due to its excellent properties and emphasizing its thermic stability [4,5]. At temperatures higher than 500–600 °C, the titanium alloys form an Oxide Layer (OL), and the oxidation process increases because the oxygen in the atmosphere diffuses faster below the OL. Additionally, oxygen interacts with the alfa-phased due to its high solubility, creating an oxygen-rich, hard, and brittle layer called “Alpha-case”, which negatively affects mechanical properties [6,7].

Ti-6Al-4V Parts fabricated by Additive Manufacturing (AM) using Laser Powder Bed Fusion (LPBF) or Electron Beam Melting (EBM) demonstrated a better performance than conventional parts in both mechanical properties and thermal stability for surgical applications [8], as a result of microstructure with fine grains [9]. Kok. et al. (2015) found that smaller thickness of an EMB part leads to better mechanical properties such as hardness, yield strength, and UT, due to higher alfa-phase related to the cooling velocity on the surface [10]. However, some post-treatments or alloying elements have been studied to optimize the performance in specific service temperatures [11,12,13].

LPBF and EBM are three dimensional (3D) AM processes which use a heat source to melt a pre-alloyed Ti-6Al-4V powder bed in a specified pattern, layer by layer, to obtain the final part [8,14,15]. Due to the high heating and cooling velocities in parts made by LPBF, the resulting microstructure contains acicular martensite (α’) with a little portion of phase β [4,16]. On the other hand, the microstructure, as a result of the EBM process, consists of a mixture of α + β type Widmanstätten due to preheating stage during the EBM [17,18,19,20]. Different types of defects reside in metallic alloys fabricated by additive manufacturing; Wysocki (2017) [16] identified that the number of pores is minor in EBM compared to LPBF, due to the preheating stages used in EBM during the printing process [8].

As reported in previous studies, superficial roughness found in as-built AM parts is related to the presence of partially melted powder and could increase the corrosion at the surface, but it does not affect the growth on the Oxygen Diffusion Zone (ODZ) [21,22,23,24,25,26]. Internal porosity and high surface roughness of alloys could lead to fatigue failure; which necessitates the need to apply post-heat treatments and surface machining in order to reduce the porosity and surface roughness of AM parts [2]. Hot Isostatic Pressing (HIP), Annealing Vacuum (AV), and Laser Shock Processing (LSP) are some of the most commonly used post-treatments to reduce the consequence of defects on the mechanical and corrosion resistance properties through the reduction in residual stress and microstructure transformations [11,24,25,26,27]. For instance, Hua et al. (2015) found that LSP reduces the oxidation velocity of alloy Ti-6Al-4V at 900 °C by temperatures of 50% [28,29,30,31].

As Ti alloys and superalloys are commonly used in high-temperature environments, corrosion tests in high temperatures are important to evaluate [32]. The kinetics behavior of oxidation is related to the oxidation stages, being categorized into three laws; (1) logarithmic law where chemisorption and the first oxygen layer formation take place, (2) parabolic law with ODZ thickening and Alpha case happening, and (3) linear law where the OL embrittlement and rupture occur.

Most of the literature articles on the Ti-6Al-4V alloys manufactured by conventional manufacturing have been dedicated to studying its tribological properties at low temperature, with applications to the energy, aeronautical or medical industries. In this context, the titanium alloys manufactured by AM, focused on the influence of process parameters on mechanical properties, microstructure and defects. Currently, very few studies have focused on the oxidation of titanium alloys manufactured by additive manufacturing. Authors such as Caballero et al., Martinez-Villafañe et al., Karlsson et al., Bermingham et al. and Casadebaigt et al. have studied the oxidation kinetics of the Ti-6Al-4V alloy with EBM processing and wire and arc additive manufacturing (WAAM) [33,34,35]. In other studies, the oxidation behavior of titanium aluminides was studied by Terner et al., Som et al. and Wang et al. [36,37,38]. One of the first studies on the oxidation kinetics of a titanium alloy (Ti-5.5Al-3.4Sn-3.0Zr-0.7Mo-0.3Si-0.4Nb-0.35Ta wt.%) manufactured by LBM was published by Zhou et al. [39]. While Liang et al. investigated the oxidation behavior of Ti-6Al-4V alloys fabricated by Selective Laser Sintering (SLS) [39,40].

Guleryuz (2009) reported that the alloy Ti-6Al-4V obeys parabolic kinetics oxidation in the range of 600–700 °C with an activation energy of 276 KJ·mol^−1^, while in higher temperatures follows a linear behavior with an activation energy of 191 KJ·mol^−1^ [20]. Dong et al. (2017) [41] reported similar values for Ti-6Al-4V alloy in the range of 199 to 281 KJ·mol^−1^ for the activation energy at temperatures between 850 and 1100 °C, obeying a parabolic law for the oxidation kinetics.

Oxidation kinetics studies of titanium alloys could find potential in the aeronautical industry applications, such as turbine blades and aircraft landing gear. The components of aircraft made with titanium alloys are exposed to different atmospheres: acid rain, marine, and high temperature (oxidizing environments).

This research aimed to study the oxidation kinetics of Ti-6Al-4V alloy by conventional and Electron Beam Additive Manufacturing. Thermogravimetric analysis was employed at temperatures of 600, 800 and 900 °C. The heating rate was 25 °C/min having an oxidation time of 24 h. By using field emission scanning electron microscope, the morphology of oxide layers and thickness was analyzed. Phases identification, before and after the oxidation process, was realized by X-ray diffraction at room temperature and hardness measurements were made in cross section.

## 2. Materials and Methods

### 2.1. Materials

Cylindrical bars of Ti-6Al-4V alloy were printed using the EBM technique and were compared to similar bars of Ti-6Al-4V alloy fabricated with conventional forging manufacturing (Table 1). The feedstock material for EBM was plasma atomized powder of Ti-6Al-4V with a nominal composition displayed in Table 1 and supplied by Arcam AB (MoIndal, Sweden). The powder was recycled and sieved multiple times between each usage and the particle size distribution ranged from 63 to 150 µm.

The samples were printed in an ARCAM Q20+ machine using a layer thickness of 90 μm and a vertical build orientation, using helium as a shielding gas.

### 2.2. Thermogravimetric Analysis

**Figure 1:**
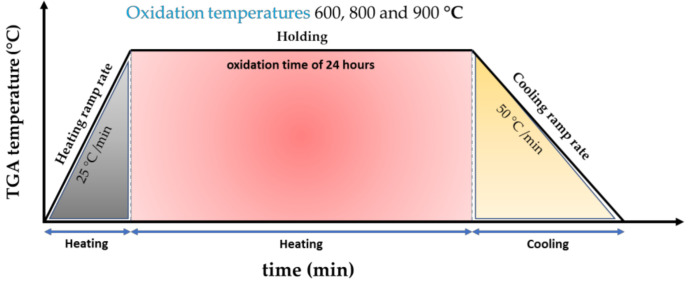
Thermogravimetric analyzer: time vs. temperature graph of heating and cooling.

Cylindrical samples of 10 mm diameter × 2 mm height were prepared according to ASTM E3-11 [42] from the as-built bar, and were ground using a 1200 grade SiC sandpaper, followed by an ultrasonic cleaning in ethanol, and then dried in air for 12 h. The grinding process was carried out to eliminate the residual superficial stress generated by EBM and the possible effect of surface roughness on oxidation behavior. A thermogravimetric analyzer (TA Instruments, New Castle, DE, USA) model TGA550 and manufactured by TA instruments, was employed to study in situ oxidation kinetics. The accuracy of the balance was 0.001 g. The samples were heated in high purity (99.9%) oxygen gas environment using a flow of 40 mL/min, at temperatures of 600, 800 and 900 °C, respectively. The heating rate was 25 °C/min with an oxidation time of 24 h (see Figure 1). The weight change was monitored over the entire exposure period with measurements conducted every second.

### 2.3. Microstructural Characterization

Microstructural characterization was performed using an optical microscope ZEISS model Axio (Jena, Germany) before thermogravimetric analysis, where the samples were polished with SiC paper and treated with 1 µm Al_2_O_3_ suspension; followed by a cleaning process with acetone and pressured air. The etching process was carried out according to ASTM E407 [43].

The thickness and morphology of Oxide layers were analyzed using a Field Emission Scanning Electron Microscope (FESEM) (ZEISS model 500 VP, Hamburg, Germany) at 20 kV. For microelemental chemical analysis and elemental mapping of the OL, the FESEM, secondary electrons (SE) detector and energy dispersive X-ray spectroscopy EDS were used.

### 2.4. X-ray Diffraction

Phase identification, after the oxidation process, was realized by X-ray diffraction (XRD) at room temperature using an X-ray Diffractometer (Malvern Panalytical Empyrean, Westborough, MA, USA), which has Cu-Kα radiation in the 2Theta range from 25 to 120 °C with a step size of 0.013°, scan step size of 29 s and constant scanning.

### 2.5. Vickers Microhardness Measurements

Microhardness measurements were realized in cross-section of the Ti-6Al-4V alloy samples using a microhardness tester (Wilson Tester 402 MVD, Lake Bluff, IL, USA) with 15 readings per sample using a 0.05 gf (load) and 15 s (dwell time).

## 3. Results and Discussion

### 3.1. OM Microstructural Analysis

**Figure 2:**
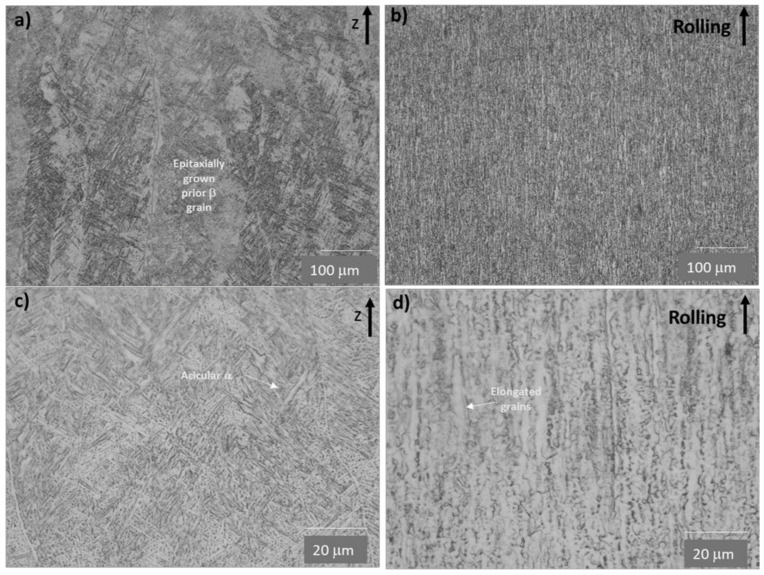
OM microstructure of as-built EBM Ti-6Al-4V (**a**,**c**) vs. conventional Ti-6Al-4V (**b**,**d**); Z and R arrows indicate the building and rolling direction on EBM and conventional samples, respectively.

Figure 2a–d shows the comparison between microstructures of as-built EBM and conventionally manufactured Ti-6Al-4V alloys before the oxidation stage. A classic Widmanstätten microstructure with fine α + β phases was observed on the EBM sample (Figure 2a,c). Some areas presented epitaxial prior β grains growing along the building direction, and inside the bright acicular α-Ti phase of different orientations and sizes. This morphology is arguable since the printing temperature range of 700–750 °C used in EBM does not reach the Martensitic transformation temperature according to the Phase diagram [21]. Furthermore, the preheating process and a slower cooling rate allowed a transformation to the phase α + β [44].

The conventional sample of Ti-6Al-4V alloy presented a typical microstructure (Figure 2b,d), of deformed α-Ti grains, elongated along the rolling direction, and surrounded by β phase [45].

### 3.2. Phase Identification by XRD

**Figure 3:**
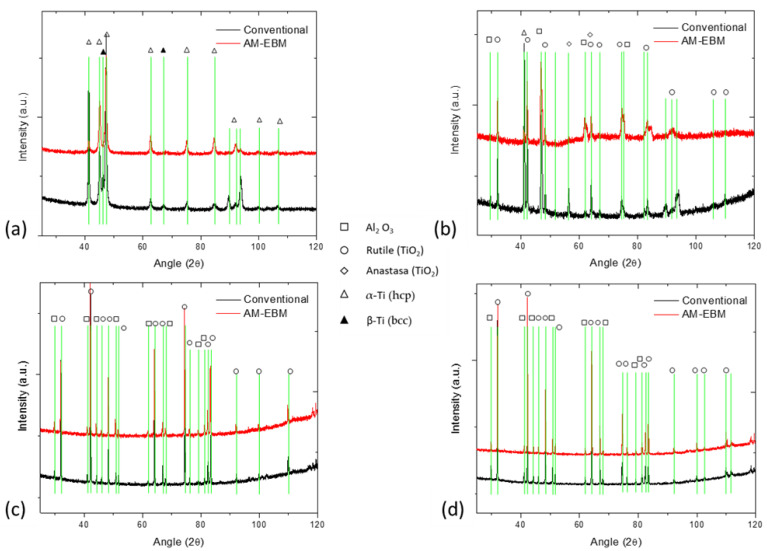
XRD patterns of the Ti-6Al-4V alloys samples (**a**) unoxidized, oxidized at (**b**) 600 °C, (**c**) 800 °C, and (**d**) 900 °C for 24 h.

Figure 3 presents the comparison of the XRD using HighScore plus 3.0 software (Malvern Panalytical, Grovewood Road, Malvern, UK), acquired for Ti-6Al-4V alloy for unoxidized and oxidized at 600, 800 and 900 °C. Figure 3a compares the XRD of EBM and conventional samples at unoxidized conditions. According to XRD, most of Bragg’s peaks belong to α-Ti with a hexagonal closed-packed (HCP) lattice (reference pattern 98-007-1963); and the intensity of the β-Ti phase peak with a body-centered cubic (BCC) structure is rather low. This peak is more clearly observed on the conventional sample due to the low volume content of this phase [46].

Substantial changes in diffraction patterns occur after the oxidation process, at 600 °C (Figure 3b), where the XRD displays Bragg peaks corresponding to three phases, the Aluminum Oxide (Al_2_O_3_) with hexagonal crystal lattice (reference pattern 98-006-1157), Titanium Oxide (TiO_2_) with two different unit cells Anastasa (reference pattern 98-000-5224), and Rutile (reference pattern 98-006-2534), and an alpha phase peak was also observed in this diffractogram [46].

As the oxidation temperature increased from 800 to 900 °C, the corresponding peaks of the Anastasa lattice disappeared, being transformed to Rutilo (reference pattern 98-002-2025, EBM and 98-006-2534, conventional samples), and along with Al_2_O_3_ (reference pattern 01-071-1683 to EBM and conventional samples) peaks become more defined. The intensity and position of the peaks are the same for both temperatures (see Figure 3c,d). This may be explained by the thermodynamics, since temperatures higher than 700 °C can be more favorable for the formation of Al_2_O_3_. Peaks corresponding to the base material (i.e., HCP or BCC) were not observed in this XRD pattern and this could be attributed to the X-ray not able to penetrate the OL and detect the base material [47]. Here the diffusion of aluminum toward OL on the surface reduced the amount of dissolved oxygen available to react with titanium [48,49,50,51]. Thus, at temperatures of 800 and 900 °C, the oxidized surface consisted mainly of rutile-type TiO_2_ and Al_2_O_3_.

### 3.3. Oxidation Kinetics: TGA and FESEM

**Figure 4:**
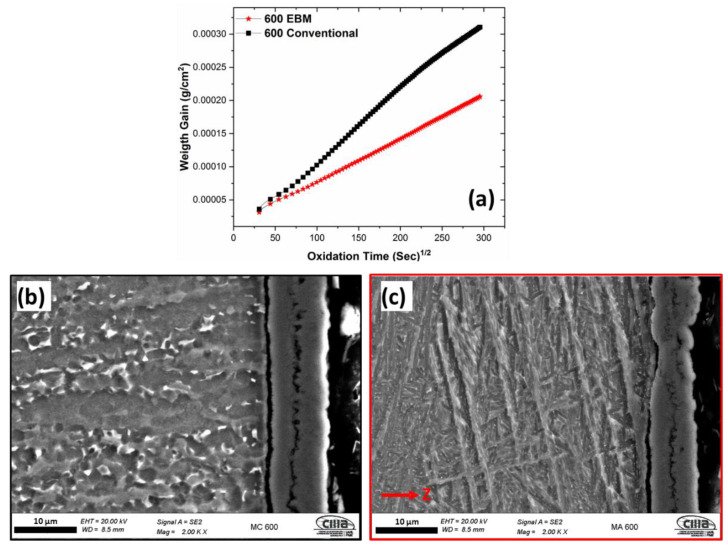
(**a**) Oxidation kinetics curves of Ti-6Al-4V alloys manufactured via EBM and conventionally; FESEM-SE morphology of the oxide layer formed at 600 °C for 24 h of (**b**) conventional and (**c**) EBM (red arrow indicates the building direction).

**Figure 5:**
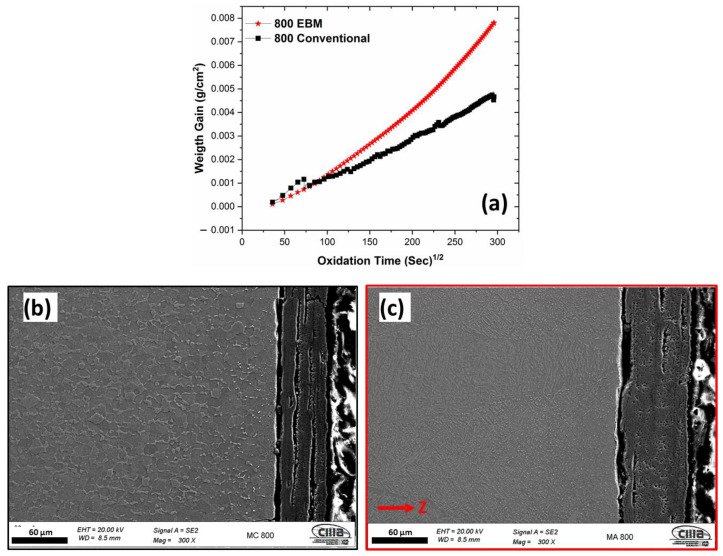
(**a**) Oxidation kinetics curves of Ti-6Al-4V alloys manufactured via EBM and conventionally; FESEM-SE morphology of the oxide layer formed at 800 °C for 24 h of (**b**) conventional and (**c**) EBM (red arrow indicates the building direction).

**Figure 6:**
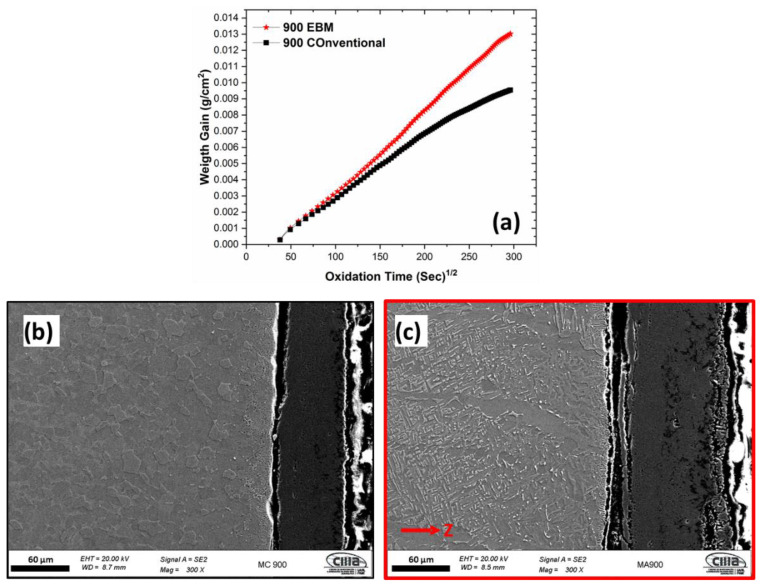
(**a**) Oxidation kinetics curves of Ti-6Al-4V alloys manufactured via EBM and conventionally; FESEM-SE morphology of the oxide layer formed at 900 °C for 24 h of (**b**) conventional and (**c**) EBM (red arrow indicates the building direction).

Figure 4, Figure 5 and Figure 6 show the dependence of EBM and conventional samples on weight gain with the oxidation time through cross-sectional FESEM morphology for 600 °C, 800 °C, and 900 °C, respectively. Samples manufactured via EBM and conventionally presented different oxidation kinetics at different times and temperatures. At temperatures of 600 °C and 900 °C, the initial oxidation stage, presents rapid increases in weight gain (around 120 min) and later the weight changes were gradual; becoming relatively stable as time increases for both EBM and conventional samples. At 800 °C, the weight gain increased rapidly in the first 120 min, however, stabilization did not occur reasonably quickly at temperatures of 600 °C and 900 °C. Therefore, this quasi-parabolic relationship may be related to the porous morphology of the oxygen layer formed at 800 °C.

Figure 4, Figure 5 and Figure 6 present the FESEM morphology of the oxygen layer formed on the top surface for each sample after 24 h of oxygen exposure. At 600 °C, a thin and porous oxygen layer was observed for both the EBM and conventional processes, nevertheless, the conventional sample demonstrated a larger thickness than the EBM sample. At 800 °C, the morphology of the oxygen layer changed to a multilayer structure with coalescence of pores and delamination on both the EBM and conventional process, but a larger thickness was observed on the EMB sample. This morphology promotes an easy penetration of oxygen into the substrate material. EBM and conventional samples showed a relatively thick and dense layer with fewer pores and limited attachment to the substrate at 900 °C of oxidation temperature. These deviations in the morphology of oxygen layers with respect to the oxidation time and temperature can be associated with the oxidation kinetics mechanisms, microstructure changes during oxidation stages, and oxidation rates [20,52,53].

Parabolic or quasi-parabolic relationships are presented for all the samples; this indicates that during the oxidation stage a parabolic law of oxidation is followed. The curves of mass change as a function of the square root of time can be fitted by a straight line, which represents the parabolic rate constant *k*, see Equation (1) [32,54]:

(1) $$\left(\frac{∆W}{A}\right)={\left(K\times t\right)}^{-2}$$

Knowing (*K*) rate constant, (Δ*W*) represents the weight gain, (*A*) denotes the surface area of each sample, and (*t*) is the oxidation time. *K* is a variable that depends on the temperature, see Arrhenius equation [32]:

(2) $$k=k_{0}\exp\left(\frac{-Q}{RT}\right)$$

The activation energy (*Q*) can be calculated from Equation (3), where: (*k*_0_) is the frequency factor, *R* is the gas constant (8.31 J/mol K) and *T* is the temperature in degrees Kelvin [32,53,54,55]:

(3) $$\log k=\log k_{0}-\left(\frac{Q}{2.30R}\right)\left(\frac{1}{T}\right)$$

**Figure 7:**
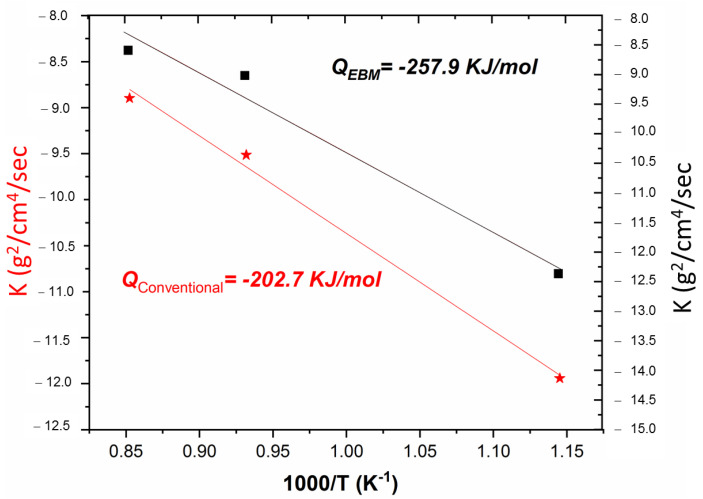
Arrhenius plot of parabolic rate constant k_0_ on the oxidation of Ti-6Al-4V alloys for conventional (red) and EBM samples (black) in the temperature range of 600–900 °C.

The linear fit between (*k*) and (1/*T*) curve for oxidation at various temperatures (show in Figure 7). The calculated values of the activation energy *Q* were 202.7 KJ/mol for titanium samples by conventional manufacturing and 257.9 KJ/mol for titanium samples by the EBM process, respectively.

**Figure 8:**
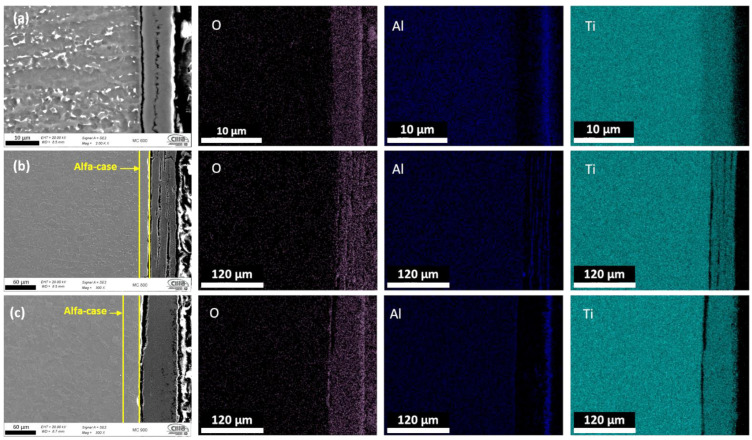
Cross section of the conventional Ti-6Al-4V alloy oxidized at (**a**) 600 °C, (**b**) 800 °C and (**c**) 900 °C after 24 h; mapping of elements by FESEM identifying the formation of oxide layers.

**Figure 9:**
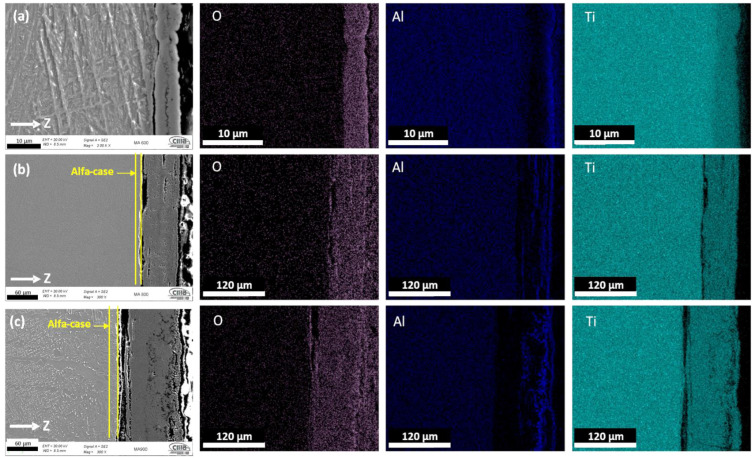
Cross section of the EBM Ti-6Al-4V alloy oxidized at (**a**) 600 °C, (**b**) 800 °C and (**c**) 900 °C after 24 h; mapping of elements by FESEM identifying the formation of oxide layers.

Figure 8 and Figure 9 show the cross-section FESEM morphology and EDX elements mappings for the Ti-6Al-4V samples manufactured conventionally and via EBM, respectively. Each figure presents the distribution of the overall element for samples oxidized at 600 °C, 800 °C, and 900 °C for 24 h. The presence and the laminate distribution of Al and O elements is observed, as the oxidation temperature increases the OL thickness increase for both conventional and EBM samples. The oxidation reaction includes the formation of OL and the diffusion of O_2_ into the base material, giving rise to the formation of the alpha-case beneath the surface oxide layer. This alpha-case greatly affects the mechanical properties of Ti alloys due to its hard and brittle properties [41,56,57]. 

At 600 °C (Figure 8a and Figure 9a), both conventional and EBM samples showed the oxidation products consisting of an inner TiO_2_ layer, corresponding to the first stage of quimisorption, where the Ti ions react with the O_2_ and form the Rutilo cell TiO_2_ [20]; and a thin Al_2_O_3_ outer layer. The morphology showed that the oxide layer exhibited good adherence to the substrate.

Conventional and EBM samples showed very similar morphology at 800 °C (Figure 8b and Figure 9b), in which alternate and porous multilayers of Al_2_O_3_ y TiO_2_ were observed. When this double-layered oxide was established, the outer Al_2_O_3_ layer developed by outward diffusion of aluminum species, while the inner layer of TiO_2_ grew by oxide ingress [40]. In Figure 9b, it can be seen that the EBM sample presented a thicker OL compared to the conventional sample (Figure 8b). 

Figure 8c and Figure 9c presents the samples oxidized at 900 °C, where the OL thickness increases as the oxidation temperature increases, but the morphology changes to a dense and thicker layer. The EBM samples presented alternate multilayers of Al_2_O_3_ y TiO_2_ and the adherence to the substrate material is very limited due to thermal coefficient differences between the base material and the oxide layers [33,58,59].

### 3.4. Vickers Microhardness Measurements

**Figure 10:**
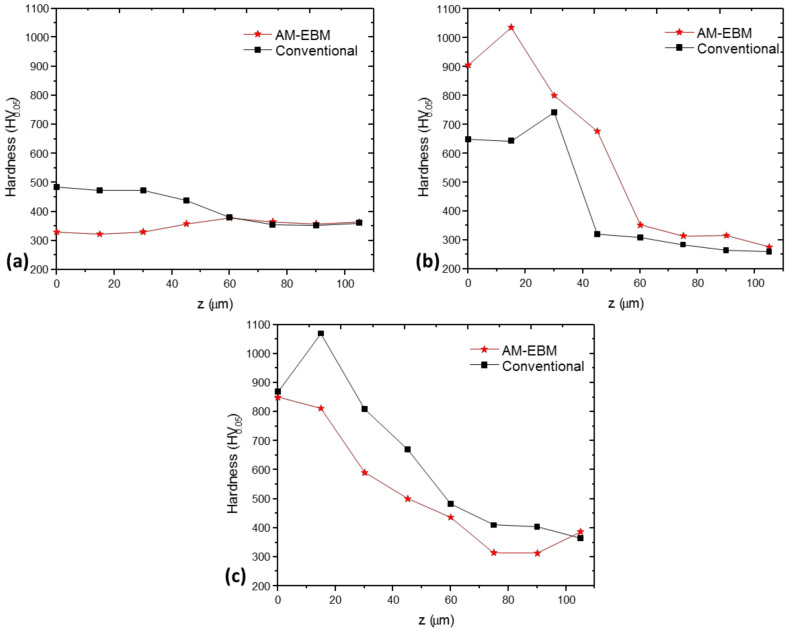
Vickers microhardness measurements (HV)–depth (X) profiles of the samples oxidized at (**a**) 600 °C, (**b**) 800 °C and (**c**) 900 °C for 24 h.

The summary of the cross-sectional hardness measurements conducted on samples oxidized at 600, 800 and 900 °C can be seen in Figure 10. At this point, it is worth mentioning that the hardness measurements were carried out starting from the top surface at 0 mm from the surface. 

Hardness values were observed to gradually decrease from the surface, on the OL to base material. Samples oxidized at 600 °C during 24 h presented hardness values in the range of 350 to 470 HV and 320 to 390 HV for the conventional and EBM samples, respectively. These hardness ranges are comparable to those reported elsewhere [54]. The differences observed could be related to the different microstructures presented by conventional and EBM samples in the unoxidized state (Figure 2) due to the low effect of the temperature (600 °C) on the microstructure.

At oxidation temperatures of 800 and 900 °C, the hardness values on the surface increased substantially but gradually decreased towards the center of the base material. The EBM sample presented higher hardness values at 800 °C compared to the conventional samples at the OL, but both samples presented similar hardness of around 700 HV near the alpha-case interface (alpha-case thicknesses of 10 µm and 20 µm, respectively), decreasing to 300 HV in the base material [52,53,54,55,56]. For oxidation temperature of 900 °C, the OL presented similar hardness values around 870 HV, decreasing gradually to 360 HV in the base material in both EBM and conventional samples (alpha-case thicknesses of 15 µm and 30 µm, respectively). Because the alpha-case thicknesses increase as the oxidation temperature increases, the hardness values remain higher before reaching the base material compared to the temperature of 800 °C [57]. The variation in hardness values has been attributed to the heterogeneity quality mainly in the OL zone [41], which is also affected by the increase in the oxidation temperature and the microstructural change produced by the diffusion of oxygen from the outside of the sample to the center. Martínez-Villafañe et al. (2003) reported values of 850 HV, however, this value was not tested at the cross-section side [51,58,59,60,61,62,63,64,65].

## 4. Conclusions

The purpose of this investigation was to determine the influence of the microstructures of the Ti-6Al-4V alloy fabricated by Electron Beam Melting and conventional forging manufacturing on the oxidation and hardness characteristics of the samples. The following summary conclusions were made based on the analysis of the results from this study:

- Oxidation rate obeyed parabolic or quasi-parabolic kinetics between 600 and 900 °C, for Ti-6Al-4V alloy fabricated by EBM is similar to conventional processed having activation energy values of Qcon = 202 kJ/mol and QEBM = 257 kJ/mol.
- The microstructure of Ti-6Al-4V fabricated by EBM is composed of columnar prior β grains elongated in the build direction and α-Ti phase. The conventional samples of Ti-6Al-4V alloy presents deformed α-Ti grains, elongated along the rolling direction, and surrounded by β phase.
- Oxidation of Ti-6Al-4V alloy in as-built and polished P1200 condition at 600, 800, and 900 °C exhibits the formation of an oxide layer formed by Anastasa and/or Rutile phase (TiO_2_) and an Alumina (Al_2_O_3_) phase at the outer scale.
- The EBM technique has a greater tendency to oxidize due to the presence of α, which has greater solubility with oxygen compared to α´.
- Cross-sectional element mapping analysis, the formation of Al_2_O_3_ and TiO_2_ multilayers were identified in both alloys. A greater thickness of the LO is identified in the detection produced by EBM with an approximate thickness of 120 µm and 65 µm for the conventional one, mainly attributed to a rougher surface, which increases the surface area of the material. The OL of both samples presents the formation of multilayers, however, in the EBM samples the presence of areas with greater porosity and less compact was demonstrated, which is consistent to that observed for the oxidation kinetics.
- Results indicate that a significant variation in the hardness change was observed, mainly in the samples exposed to 800 and 900 °C; reaching values of 900 HV (EBM) and 700 HV (conventional) at 800 °C, and 1000 HV (EBM) and 850 HV (conventional) at 900 °C. At 600 °C, no significant effect on hardness was observed due to the low effect of temperature on the microstructural change. At 800 and 900 °C, on the contrary, a significant effect was observed with the formation of the OL and the change in the microstructure alpha-case.

## Figures and Tables

**Table 1:** Chemical Composition of the Ti-6Al-4V alloy (wt.%).

Process	Elements								
	Al	V	Fe	O	C	N	H	Y	Ti
EBM powder	6.68	3.78	0.19	0.13	0.02	0.02	0.002	<0.001	Balance
Conventional Part	7.14	4.03	0.21	_	_	_	_	_	Balance
Powder by ASTM F2924	5.5–6.75	3.5–4.5	0.3	0.2	0.08	0.05	0.015	0.005	Balance

## Data Availability

Not applicable.
